# The Adherence Conundrum of Sleep Restriction Therapy for Insomnia

**DOI:** 10.1111/jsr.70339

**Published:** 2026-04-01

**Authors:** Mathilde I. Looman, Mona M. Klau, Tim M. Schoenmakers, Jan H. Kamphuis, Tessa F. Blanken, Jaap Lancee

**Affiliations:** ^1^ Department of Clinical Psychology University of Amsterdam Amsterdam the Netherlands; ^2^ Department of Psychological Methods University of Amsterdam Amsterdam the Netherlands

## Abstract

While time in bed restriction is a core treatment mechanism of Sleep Restriction Therapy (SRT), empirical research has not yielded a relationship between insomnia improvement and adherence to the prescribed time in bed. We coin this discrepancy the adherence conundrum of SRT. Understanding this adherence conundrum is clinically important, as stringent time in bed restriction makes SRT particularly demanding for patients. We propose that one important aspect has been overlooked in adherence studies and could potentially obscure an adherence‐effect relationship: the achieved dose of time in bed restriction. To explore this possibility, we conducted a secondary analysis of a randomised controlled trial in which adults with insomnia received 6 weeks of SRT (*N* = 54). As did previous studies, we found that adherence to the prescribed time in bed did not predict insomnia severity, *β* = −0.03, *t* = 1.66, *p* = 0.10. Contrary to expectations, controlling for achieved dose did not alter this finding, *β* = −0.03, *t* = 1.48, *p* = 0.14. As most participants achieved a large dose of time in bed restriction, a ceiling effect may be operating, beyond which adherence may offer little incremental benefit. We call for research into the adherence conundrum and the optimal prescribed time in bed that balances tolerability and efficacy. Such research will be essential to guiding how strictly the rules of SRT need to be enforced in clinical practice.

**Trial Registration:** Sleep Restriction Treatment for Insomnia. https://clinicaltrials.gov/study/NCT05548907

## 1 Introduction

Sleep Restriction Therapy (SRT) is an effective treatment for insomnia (Maurer et al. 2021). At the start of this behavioural intervention, the time in bed (TIB) is restricted to match the self‐reported total sleep time, based on sleep diary averages (Spielman et al. 1987). Implementing TIB restriction is often experienced as challenging (Kyle et al. 2011). Patients may be reluctant to shorten their bedtimes because of feared consequences, such as increased daytime sleepiness. As a result, therapists and patients may negotiate adjustments to the prescribed TIB, or patients may not fully adhere to the prescribed schedule.

At the same time, TIB restriction is assumed to be a core mechanism of change in SRT. Theoretically, restricting TIB is hypothesised to improve insomnia by increasing homeostatic sleep drive and decreasing arousal (Maurer et al. 2018). Restricting TIB may also challenge dysfunctional cognitions (e.g., “I need at least 8 h of sleep”) and preclude safety behaviours (e.g., staying in bed longer to compensate for sleep loss). Empirical evidence from dismantling trials supports the mechanistic role of TIB restriction: combining regularising and restricting TIB is more effective in reducing insomnia than regularising alone (Maurer et al. 2020). Moreover, stronger TIB restriction at treatment onset has been associated with faster symptom improvement compared to more gradual restriction, as in sleep compression therapy (Rosén et al. 2023; Jernelöv et al. 2025).

Based on the notion that TIB restriction is a mechanism of change of SRT, it has been assumed that adherence to TIB restriction is important; those with better adherence are expected to benefit more from treatment (Mellor et al. 2022). However, previous research does not consistently find that TIB adherence is related to treatment effect (Agnew et al. 2021). This inconsistency may partly reflect heterogeneous adherence measures, which range from clinician ratings (potentially biased by treatment response) to global CBT‐I patient ratings that do not specifically assess SRT (Agnew et al. 2021). To standardise operationalisations, researchers are recommended to report the raw minute deviation between the prescribed and actual time in bed based on sleep diary data (Agnew et al. 2021; Steinmetz et al. 2023). Yet also with this operationalisation, adherence did not predict insomnia severity (Steinmetz et al. 2023). We are thus faced with a conundrum: on the one hand, theory and evidence from dismantling studies suggest that TIB restriction is a core mechanism of SRT; on the other hand, SRT studies do not support a relation between TIB restriction adherence and treatment effect. What to make of this discrepancy?

A factor that may obscure the relationship between TIB and treatment efficacy is that adherence studies generally do not consider the *achieved dose* of TIB restriction. In medical research, dose (the actual amount of medication taken) is often considered alongside adherence (compliance with the prescription), based on the premise that dose mechanistically affects response (a dose–response relationship; Redelmeier and Zipursky 2023). In SRT, for example, two individuals may both deviate 60 min from their prescribed TIB yet differ substantially in their achieved dose: one may have reduced TIB by 120 min instead of the prescribed 180 min, while the other implemented zero restriction instead of the prescribed 60 min. While their adherence deviation scores are identical (60 min), their achieved dose of TIB restriction differs markedly (120 vs. 0 min). Conceivably, the achieved dose of TIB restriction influences treatment effects, obscuring adherence findings in SRT studies. To explore this possibility, we conducted a secondary analysis on data from a randomised controlled trial on telephone‐guided SRT (Looman et al. 2025).

## 2 Methods

Participants (*N* = 54) were adults with insomnia (Insomnia Severity Index; ISI ≥ 10) who completed a daily online version of the Consensus Sleep Diary (Carney et al. 2012) throughout 6 weeks of SRT and completed the ISI (Morin et al. 2011) at pre‐ and posttest. Of the 76 participants assigned to SRT in the parent trial, we excluded those who dropped out of the trial (*n* = 19) and those who completed fewer than 10 sleep diary entries in the first 14 treatment days (*n* = 3). We focused on the first treatment weeks, during which adherence is suggested to be most difficult and critical (Kyle et al. 2011). We assessed whether higher adherence to TIB restriction was associated with a larger treatment effect on insomnia severity from pre‐ to post‐treatment, both before and after controlling for the achieved dose of TIB restriction. We measured TIB adherence as the raw minute deviation from the prescribed TIB based on Spielman's rules (Spielman et al. 2010) during the initial treatment phase (i.e., the first 14 days). Table 1 presents the study variables, their calculations, and descriptive statistics.

**TABLE 1:** Study variable calculations and descriptives.

Variable	*M*, (SD)	Observed range	Formula
Pre‐test insomnia severity	18 (3)	10, 25	
Post‐test insomnia severity	9 (5)	0, 25	
Achieved dose (minutes)	93 (43)	9, 208	$D_{a}=\mathrm{TI}B_{\mathrm{PRE}}-\frac{\mathrm{TI}B_{W1}\;+\;\mathrm{TI}B_{W2}}{2}$
Prescribed dose (minutes)	122 (43)	53, 225	$D_{p}=\mathrm{TI}B_{\mathrm{PRE}}-\frac{\max\;\left(\mathrm{TS}T_{\mathrm{PRE}},\;300\right)\;+\;\max\;\left({\mathrm{TST}}_{\mathrm{PRE}}\;+\;\mathrm{Adj},\;300\right)}{2}$
Adherence (minutes)	−29 (31)	−126, 40	$A=D_{a}-D_{p}$

*Note:* Adj = adjustment. If SE_1_ < 85%, then adjustment is −15; if 85% < SE_1_ < 90%, then adjustment is 0; if SE_1_ > 90%, then adjustment is +15, with a minimum of 5 h (300 min). Subscript w1 refers to days 1 to 7 of treatment (week 1), and w2 refers to days 8 to 14 of treatment (week 2).

## 3 Results

When controlling for pre‐treatment insomnia severity, adherence did not significantly predict posttest insomnia severity, *β* = −0.03, *t* = 1.66, *p* = 0.10. Adherence also did not predict posttest insomnia severity when controlling for the achieved dose, *β* = −0.03, *t* = 1.48, *p* = 0.14. Adherence and TIB restriction dose were moderately positively correlated, *r* = 0.36, *p* = 0.01, indicating that they are related yet distinct constructs. Together, these findings provide no evidence that adherence to TIB restriction relates to treatment response, even after considering the achieved dose.

## 4 Discussion

Our findings highlight the ongoing adherence conundrum of SRT: on the one hand, our null findings align with earlier studies that did not detect an adherence‐response relationship (Agnew et al. 2021; Steinmetz et al. 2023). On the other hand, our results are not in line with mechanistic accounts and dismantling evidence that highlight TIB restriction as central to change (Maurer et al. 2018, 2021). This raises important questions for both clinical practice and the treatment mechanism of SRT.

Regarding clinical practice: how strictly should the rules of restriction be enforced? If better adherence relates to improved outcome, the answer would be clear: strict enforcement is crucial. However, our results, together with prior studies, suggest that this may not be the case. This may warrant investigating if the rules of SRT could be slightly relaxed to make the intervention more feasible and potentially shield against current dropout. Sleep compression therapy addresses this aim (Lichstein 1988), adopting a milder approach with an initial TIB reduction of 20% of total wake time, instead of 100% as in SRT. However, SCT failed to show non‐inferiority to SRT (Jernelöv et al. 2025). The optimal TIB restriction that balances tolerability vs. efficacy may lie somewhere between these two approaches. Clearly, this conjecture needs to be evaluated in a controlled experimental study.

With respect to the treatment mechanism of SRT: how can these null findings be reconciled with the assumption that TIB restriction is a core mechanism of change in SRT? One hypothesis to consider is a ceiling or plateau effect; once a sufficient dose of restriction is achieved, additional precision in following the rules may add little incremental benefit. If this holds, the lack of an adherence‐effect relationship in our sample could be explained because most participants achieved relatively large TIB reductions, 95% CI [82 – 105] minutes. We could not adequately test for a ceiling effect as this would require sufficient variability in low achieved TIB dose (< 50 min). It is possible that those with a low achieved dose dropped out of the trial. As most trial dropouts completed no assessments beyond the pre‐test, we could not reliably impute their missing data. Hence, testing the ceiling effect remains an important target for further research.

Other aspects to consider are that this study, consistent with previous work, utilised sleep‐diary data to assess adherence; while clinically valuable, it would be informative to examine adherence using physiological measures such as actigraphy. Moreover, reduced variability in bed‐ and risetimes is another potential mechanism of SRT and may serve as an alternative measure of adherence (Maurer et al. 2021). In addition, adherence and response may be influenced by individual difference factors. For instance, individual sleep needs may affect both ease of adherence and response to a given dose. Finally, this study only had sufficient power to detect medium or large adherence–response effects. Retesting in larger samples is needed to detect possible subtle effects of adherence.

## 5 Conclusion

The role of TIB adherence in relation to SRT's treatment effect remains unclear and should not be assumed at face value. Our report does not resolve the conundrum of TIB restriction in SRT but, in fact, underscores it. We call for studies to examine both adherence and its relationship with treatment effect, taking the achieved dose of restriction into account. Such research will help develop evidence‐based guidelines for how strictly adherence should be emphasised and negotiated in clinical practice.

## Author Contributions

**Mathilde I. Looman:** conceptualisation, data curation, formal analysis, investigation, methodology, project administration, resources, software, validation, visualisation, writing – original draft, writing – review and editing. **Mona M. Klau:** conceptualisation, data curation, formal analysis, methodology, project administration, resources, software, validation, visualisation, writing – original draft, writing – review and editing. **Tim M. Schoenmakers:** funding acquisition, investigation, project administration, resources, writing – review and editing. **Jan H. Kamphuis:** funding acquisition, project administration, resources, validation, writing – review and editing. **Tessa F. Blanken:** conceptualisation, funding acquisition, investigation, methodology, project administration, resources, writing – original draft, writing – review and editing. **Jaap Lancee:** conceptualisation, funding acquisition, investigation, methodology, project administration, resources, writing – original draft, writing – review and editing.

## Funding

This work is funded by an internal research grant from the University of Amsterdam. The funder had no role in the design, data collection, data analysis, or reporting of the study.

## Ethics Statement

This study was reviewed and approved by the ethical committee of the University of Amsterdam (2022‐CP‐15342).

## Consent

All participants provided written informed consent.

## Conflicts of Interest

The authors declare no conflicts of interest.

## Data Availability

As public data sharing was not part of the informed consent process, the data supporting the findings of this study are not publicly available. The data are available from the corresponding author upon reasonable request.
